# Individual variation in migratory movements of chinstrap penguins leads to widespread occupancy of ice-free winter habitats over the continental shelf and deep ocean basins of the Southern Ocean

**DOI:** 10.1371/journal.pone.0226207

**Published:** 2019-12-10

**Authors:** Jefferson T. Hinke, Maria M. Santos, Malgorzata Korczak-Abshire, Gennadi Milinevsky, George M. Watters

**Affiliations:** 1 Antarctic Ecosystem Research Division, Southwest Fisheries Science Center, National Marine Fisheries Service, National Oceanic and Atmospheric Administration, La Jolla, California, United States of America; 2 Departamento Biología de Predadores Tope, Instituto Antártico Argentino, San Martín, Argentina; 3 Laboratorios Anexos, Facultad de Ciencias Naturales y Museo, Universidad Nacional de La Plata, La Plata, Argentina; 4 Institute of Biochemistry and Biophysics, Polish Academy of Sciences, Warsaw, Poland; 5 National Antarctic Scientific Center of Ukraine, Kyiv, Ukraine; MARE – Marine and Environmental Sciences Centre, PORTUGAL

## Abstract

A goal of tracking migratory animals is to characterize the habitats they use and to interpret population processes with respect to conditions experienced en route to, and within, overwintering areas. For migratory seabirds with broad breeding ranges, inferring population-level effects of environmental conditions that are experienced during migratory periods would benefit by directly comparing how birds from different breeding aggregations disperse, characterizing the physical conditions of areas they use, and determining whether they occupy shared foraging areas. We therefore tracked 41 adult and juvenile chinstrap penguins (*Pygoscelis antarctica*) from three breeding locations in the northern Antarctic Peninsula region during the austral winter of 2017. The satellite tracking data revealed overlap of individuals over continental shelf areas during autumn months (Mar-May), shared outbound corridors that track the southern Antarctic circumpolar current front, followed by occupancy of progressively colder, deeper, and ice-free waters that spanned the entire western hemisphere south of the Polar Front. Despite broadly similar physical environments used by individuals from different colonies, the proportion of birds from each colony that remained within 500km of their colony was positively correlated with their local population trends. This suggests that local migration strategies near the Antarctic Peninsula may benefit breeding populations. However, the magnitude of inter-colony and intra-colony overlap was generally low given the broad scale of habitats occupied. High individual variation in winter movements suggests that habitat selection among chinstrap penguins is more opportunistic, without clear colony-specific preference for fine-scale foraging hotspots. Mixing of individuals from multiple colonies across broad regions of the Southern Ocean would expose chinstrap penguins from the Antarctic Peninsula to a shared environmental experience that helps explain the regional decline in their abundance.

## Introduction

Migration links disjunct habitats that are vital to the success of many species. Regular movement between, and occupation of, such different areas is generally thought to arise because fitness benefits accrue in different habitats at different times. For example, conditions encountered within foraging areas during the non-breeding period (hereafter winter) are hypothesized to affect survival and future reproductive success of many migratory animals [1,2], ultimately affecting population trends [3]. Thus, one goal of tracking studies is to characterize the habitats that are used by animals in the course of their movements and to interpret population processes with respect to the range of conditions experienced.

While the distribution of summer and winter habitats used by some species are well known and leveraged effectively in conservation and management efforts [4], information on the seasonal distributions of other species is less well defined. This is particularly true for species with broad, but disjointed breeding distributions, like many seabirds that are often confined to isolated breeding locations during summer but disperse broadly during winter. For such widely ranging seabirds, inferring the effects of winter environmental conditions on population trends would benefit from improved understanding of how animals from different breeding aggregations disperse, whether they share common foraging areas during the winter, and to assess potential risks in shared foraging areas [5,6].

Here, we consider the chinstrap penguin (*Pygoscelis antarctica*), a migratory seabird with a circumpolar breeding distribution that is centered primarily on the island archipelagos in the south Atlantic sector of the Southern Ocean. Worldwide, the IUCN rates the conservation status of chinstrap penguins as ‘least concern’ with a declining population currently estimated around 8 million mature individuals [7]. A decline in adult abundance is the predominant trend observed at breeding colonies near the Antarctic Peninsula and South Shetland Islands [8]. Such regional declines are hypothesized to derive from shared environmental drivers during winter [9,10].

Prior telemetry studies of chinstrap penguins during winter have revealed a range of individual-level movement patterns, from retention near natal breeding sites to rapid and long-distance dispersal to remote areas [11–15]. Stable isotope analysis of feather tissues that are grown during the migratory period further suggest that colony location may influence the primary direction (east or west from natal colonies) of migration on a longitudinal basis [16], with birds from the Antarctic Peninsula region generally tending westward. Such large-scale gradients in movement patterns suggest that inter-colony variation in chinstrap population trends could be related to differences in wintering areas used by birds from particular colonies. Alternatively, high intra-colony variation in individual movement patterns may facilitate shared environmental experiences across colonies. A test of these alternative hypotheses requires a concurrent, multi-colony tracking study at sites with contrasting population trends, preferably including multiple life stages to assess differences between adult and juvenile birds [6,17,18]. Here, we report on simultaneous releases of chinstrap penguins instrumented with satellite telemetry devices from three breeding colonies with contrasting population trends [19,20,21] in the northern Antarctic Peninsula region during the winter of 2017.

We focus our analysis on the spatial distribution of the chinstrap penguins during winter and the environmental conditions encountered by them: sea ice, sea-surface temperature, and surface currents. Chinstrap penguins are considered an ice-avoiding species, generally being observed in open waters seaward of the edge of sea ice [22], thus ice edges may be important habitat delimiters. Sea-surface temperatures and currents are major factors that delineate oceanographic fronts in the Southern Ocean [23] and help define foraging grounds for numerous species in the Southern Ocean [24]. Furthermore, temperatures and frontal features can affect the distribution and abundance of prey organisms like Antarctic krill (*Euphausia superba*), fish, and squid, which form the majority of the prey consumed by chinstrap penguins [25]. Our aims are, thus, to 1) describe movement patterns from multiple breeding colonies in the Antarctic Peninsula region, 2) characterize the physical marine habitats that are used by adult and juvenile chinstrap penguins during the winter period, and 3) assess the extent of inter-colony and intra-colony overlap in habitat use during winter on a large (e.g., basin-level) and a small (e.g., individual) scales.

## Materials and methods

### Ethics statement

Research was permitted under U.S. Antarctic Conservation Act Permits (Permit #2017–012), the Polish Permitting Authority IBB PAS (Permit #05/2016), and the Argentine Dirección Nacional del Antártico Environmental Office (Permit 2017–010). Field protocols were approved by the University of California San Diego Institutional Animal Care and Use Committee (S05480).

### Tagging sites

**Fig 1:**
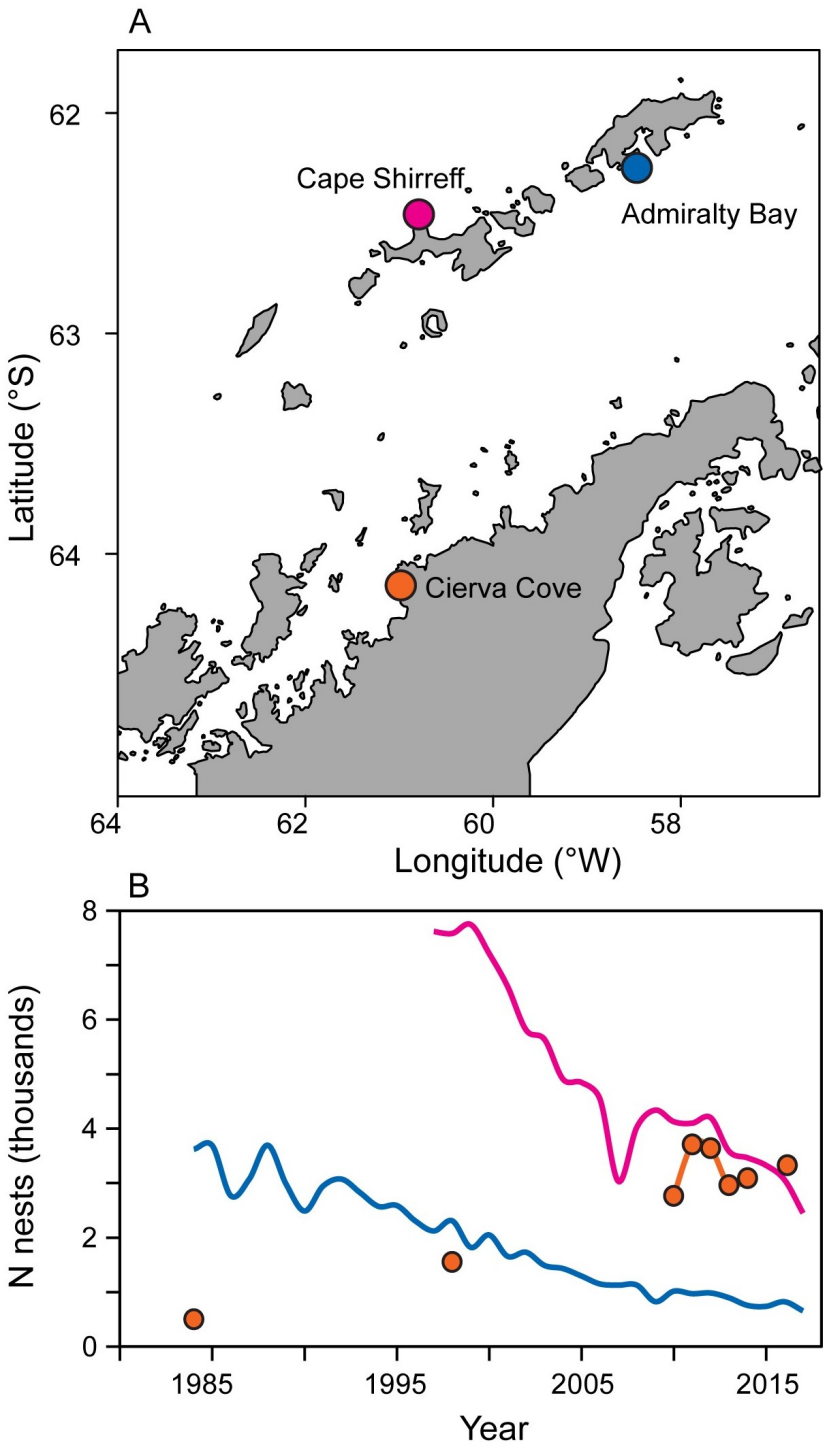
Location and breeding population sizes at each tagging site. A) Location of tagging sites. B) Population sizes over time at each tagging site. Colors indicating Admiralty Bay (blue), Cape Shirreff (magenta), and Cierva Cove (orange) are matched across panels.

We conducted our study at Cape Shirreff and Admiralty Bay, in the South Shetland Islands and at Cierva Cove, along the western Antarctic Peninsula (Fig 1). Over the last 30 years, breeding populations of chinstrap penguins at Cape Shirreff (Livingston Island) and Admiralty Bay (King George Island) have declined [19], consistent with the predominant population trend observed in the northern Antarctic Peninsula region [8,20], while the population at Cierva Cove, in the northern Gerlache Strait, has increased [21].

### Instrumentation

Post-molt adult and fully-fledged juvenile chinstrap penguins were captured for instrumentation between 18 February 2017 and 9 March 2017 (Table 1). We assume, based on opportunistic tracking of pre-molt foraging trips of chinstrap adults [15] and observations of molting individuals marked with flipper bands (JTH, pers obs), that all adults tagged in this study were occupants of the colony in which they molted. In total, 35 adults were fitted with Wildlife Computers Spot-275 satellite transmitters (86 x 17 x 18mm, 38g), and 10 juveniles were fitted with Sirtrack Kiwisat-202 K2G-173A satellite transmitters (60 x 27 x 17mm, 34g). Transmitters were affixed to feathers along the midline of the spine using either quickset epoxy or cyanoacrylate glue. Small plastic cable ties were threaded through underlying feathers and closed over the top of the tag as an additional fastener. All transmitters were scheduled to transmit daily for six consecutive hours (12:00–18:00 UTC) until battery failure. No tracked birds were identified in the study colonies in the following breeding season.

**Table 1:** Summary of satellite-transmitter deployment information.

Tagging location	Longitude (°W)	Latitude (°S)	Age class	N. deployed	Date deployed	Viable data sets
Admiralty Bay	58.469	62.236	Adult	10	3/9/2017	9
Cape Shirreff	60.789	62.46	Adult	15	2/19/2017	15
			Juvenile	5	2/18/2017	4
Cierva Cove	60.984	64.143	Adult	15	2/25/2017	10
			Juvenile	5	2/25/2017	3

Viable data sets are those with ≥ 7 d of data.

### Analysis of telemetry data

The raw location data collected by the ARGOS satellite system were processed to remove erroneous location estimates as indicated by “Z” quality codes, unspecified ellipse errors, and as indicated by speed filters with a conservative constant swim speed of 2.5 m/s. We arbitrarily restricted our data set to deployments with at least one week of location estimates to minimize over-representing near-colony habitat use immediately following molt or fledging. This restriction removed 15% of all adult tracks and 30% of all juvenile tracks (Table 1). All retained tracks were then smoothed to a 2-hr interval with a state space model [26] using the R [27] package ‘crawl’ [28]. The resulting model fit was used to generate 100 alternative tracks for each deployment with sample locations taken every 2 hours from tag release to tag failure. The alternative tracks were pooled and mapped to hexagonal polygons with centroids spaced 25 km apart (area ≈ 541 km^2^) to produce habitat utilization distributions (HUD) using the R package ‘crawlr’ [29]. This relatively small spatial scale approximates daily movements of chinstrap penguins during the summer breeding season [15]. Monthly estimates of inter- and intra-colony overlap were calculated from these HUDs by computing the total area where at least two individuals co-occurred relative to the total area occupied by all tracks.

To assess movement of birds on a larger scale, we classified each deployment into western, local or eastern bins to assess the frequency of different, large-scale migration patterns within colonies. The classification for each deployment was based on the net direction traveled and maximum distance achieved from its tagging location. We assigned a track to the local bin if it remained within a 500 km radius of its tagging location. This arbitrary radius was based on assessments of the maximum distance attained from each tag’s origin (S1 Fig). It is evident that some long-distance migrants remain within 500km for some time, but ultimately initiate directed movement away from local areas within the first 12 weeks of deployment. Over that same period, none of the local birds demonstrated such tendency to initiate sustained directed movement. From that perspective, the 500km delimiter distinguishes the range of behaviors exhibited by long-distance migrants from those with local tendencies. Tracks assigned to each movement bin are illustrated in S2 Fig and we note that at least two tracks from each directional bin exceed 13 weeks. Note also that birds in the local bin also had the shortest average deployment durations, particularly among the juvenile penguins (Table 2).

**Table 2:** Summary of tracking data from all deployments.

Tagging location	Direction	Stage	N tags	Mean (range) duration (d)	Mean (range) maximum distance (km)	Mean daily swim speed (m/s)	Max daily swim speed (m/s)
Admiralty Bay	East	A	2	109 (94–123)	1221 (890–1552)	0.16 ± 0.16	0.48
	Local	A	6	46 (19–124)	152 (22–480)	0.05 ± 0.05	0.19
	West	A	1	104	1475	0.16 ± 0.16	0.38
Cape Shirreff	East	J	1	49	2183	0.52 ± 0.14	0.65
	Local	A	7	65 (9–198)	215 (25–493)	0.08 ± 0.07	0.26
	Local	J	3	9 (8–10)	119 (60–174)	0.13 ± 0.05	0.17
	West	A	8	143 (46–254)	1691 (629–4124)	0.17 ± 0.14	0.52
Cierva Cove	Local	A	8	66 (19–140)	199 (21–401)	0.04 ± 0.04	0.16
	Local	J	3	12 (7–20)	180 (111–223)	0.16 ± 0.11	0.32
	West	A	2	161 (135–187)	2789 (799–4779)	0.26 ± 0.28	0.95

Mean and range of deployment durations, maximum distances reached, and sustained swim speeds for adult (A) and juvenile (J) stages from each tagging location at Admiralty Bay, Cape Shirreff, and Cierva Cove. Swim speed was calculated from the mean of net distances moved per month, weighted by the number of days tracked in each month.

### Physical environmental conditions

Average, monthly environmental conditions encountered during the study period were extracted from data layers characterizing habitat covariates (see below) using the areal extent of each monthly HUD. Monthly indices were selected to provide conservative estimates of environmental conditions given inherent uncertainty in location estimates and to limit over-interpretation of environmental conditions experienced by the few animals tracked in such remote locations. The covariates considered here included bathymetry, zonal (west-to-east) surface currents, sea-surface temperature (SST), and sea-ice concentration (SIC). We used the bathymetry data from ETOPO1 [30] to extract bottom depths. Zonal surface currents were extracted from output of the Ocean Surface Currents Analyses Real-time (OSCAR) model, resolved monthly on a 1/3° grid [31,32]. Sea-surface temperatures were extracted from monthly MODIS Aqua Level 3 SST data resolved on a 9 km grid [33,34]. Similarly, monthly SIC data were extracted from NOAA/NSIDC Climate Data Record of Passive Microwave Sea Ice Concentration, Version 3 [35,36] resolved on a 25 km grid. We extracted the mean environmental value within each hexagonal unit in the HUD using the ‘raster’ package [37] in R.

We also matched raw position estimates with daily estimates of SST and SIC. These along-track indices were created to complement the monthly composites from the HUDs and to examine near real-time experience of SST and SIC that the monthly composites may smooth. We used daily Multi-scale Ultra-high Resolution (MUR) SST resolved on a 1 km grid [38] and daily SIC data from the EUMETSAT Ocean and Sea Ice Satellite Application Facility (OSI-SAF), interpolated from a native 10 km grid to a 1 km grid.

Finally, we used the locations of the Polar Front (PF), southern Antarctic circumpolar current front (SACCF), and southern boundary of the Antarctic circumpolar current (SBACC) [23], available in R package ‘orsifronts’, to help delimit the general oceanic areas used by the penguins.

### Estimation of habitat area

Given the wide range of ocean areas used by the tagged penguins (S2 Fig), we created a 2-part index of monthly habitat availability from 180°W to 0°W. The first part measures area of open water habitat between the 5% SIC isocline (or coastline if ice is absent) and the 2°C isotherm to encompass the distribution of SST encountered by chinstraps here and consistent with results from a prior study of their migration [14]. The second part measures the area of the marginal ice zone (MIZ), measured here as the area between the 5% to the 50% SIC isoclines, consistent with observed concentrations of sea ice encompassed by the birds’ HUDs. The SIC and SST isoclines were converted to spatial polygons using the ‘sp’ package [39], and the total area of the polygon(s) defined by the isoclines was calculated using the ‘rgeos’ package [40]. Habitat areas were then calculated by differencing the polygons and any intersection of those polygons with a land mask derived from the Global Self-consistent, Hierarchical, High-resolution Geography Database [41].

## Results

Data with ≥1 week of at-sea position estimates were collected from 34 adults and 7 juveniles, representing 90% of all deployments (Table 1). On average, the positions of adult and juvenile penguins were respectively reported for 90 days (range: 9–254 days) and 16 days (range: 7–49 days; Table 2). Tags on adults reported 6 ± 2.4 (sd) location estimates per day, while tags on juveniles provided 3.4 ± 1.8 (sd) location estimates per day. Maximum great-circle distances from natal colonies averaged 795 km (range: 21–4779 km) for adults and 439 km (range: 60–2182 km) for juveniles (Table 2).

Directional movements varied by life stage and colony, with no eastward movement recorded from Cierva Cove and no westward movement observed among juveniles (S2 Fig). Most tracks were classified as local. The proportion of birds that remained within 500 km of their natal colony was highest at Cierva Cove, where populations have increased, and lowest at Cape Shirreff, where populations have declined (Fig 1, Table 2). A mixed-effect model [42] with each individual treated as a random effect suggested that average monthly swim speeds did not vary by tagging site, but did vary by direction of travel, with the local migrants exhibiting generally slower mean speeds than east-bound and west-bound migrants (Table 2, χ^2^ = 22.4, df = 2, p<0.001). Due to small sample sizes and short durations of juvenile penguin deployments (Tables 1 and 2), and the general overlap of juveniles with adult tracks (S2 Fig), all tracks were pooled for further analysis.

**Fig 2:**
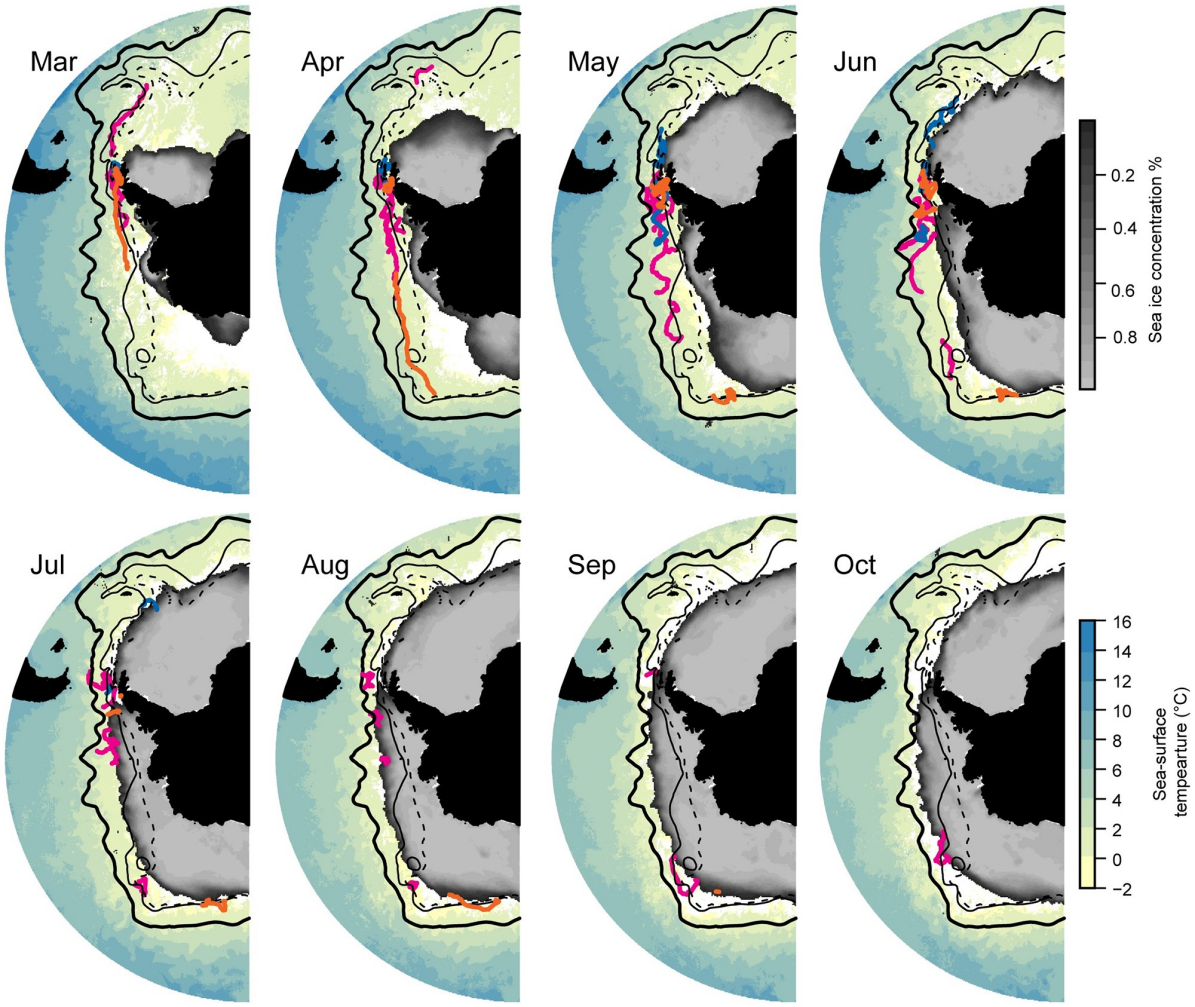
Monthly maps of penguin positions and environmental conditions. Monthly at-sea locations of all chinstrap penguins originating from Admiralty Bay (blue), Cape Shirreff (magenta), and Cierva Cove (orange) overlaid on mean monthly sea-surface temperatures and mean monthly sea-ice concentrations, March—October, 2017. The Polar Front (thick solid line), southern Antarctic circumpolar current front (thin solid line) and southern boundary of the Antarctic circumpolar current (thin dashed line) are plotted for reference. White areas indicate no data.

**Fig 3:**
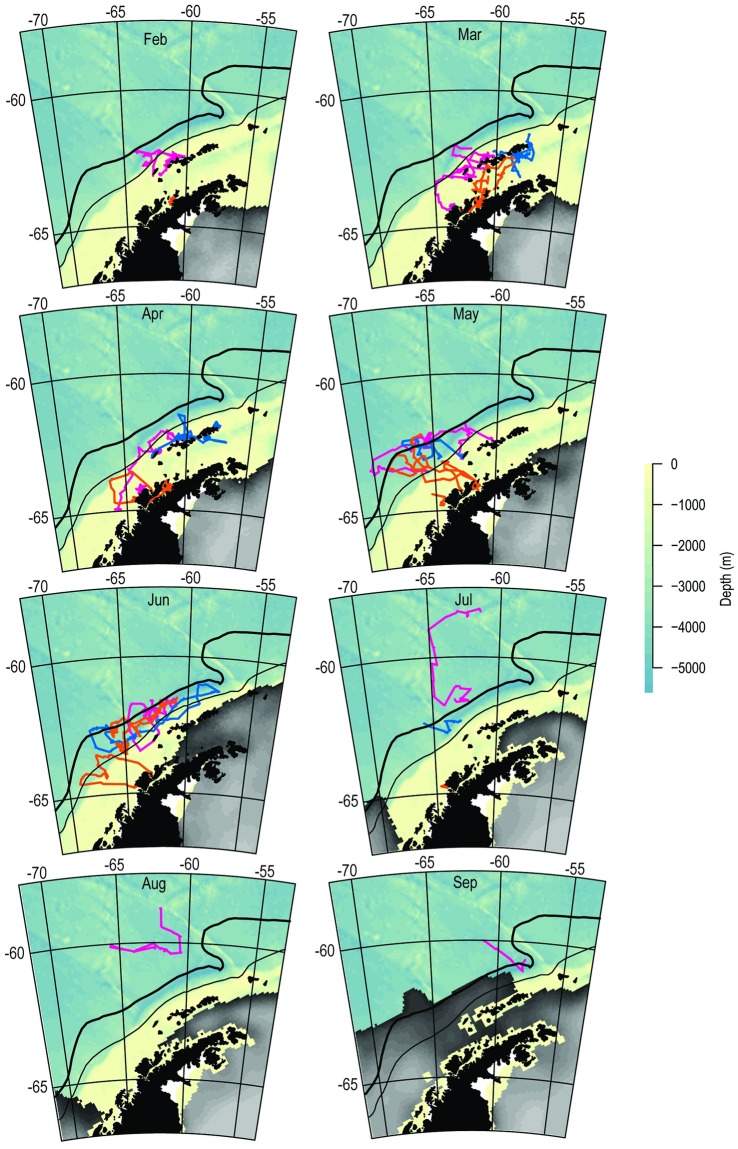
Monthly maps of penguins positions for ‘local’ individuals. Monthly at-sea locations for all ‘local’ chinstrap penguins originating from, and remaining within 500km of, Admiralty Bay (blue), Cape Shirreff (magenta), and Cierva Cove (orange). Tracks are overlaid on bathymetry and mean monthly sea-ice concentrations, February—October, 2017. The Polar Front (thick solid line), southern Antarctic circumpolar current front (thin solid line) and southern boundary of the Antarctic circumpolar current (thin dashed line) are plotted for reference. White areas indicate no data.

**Fig 4:**
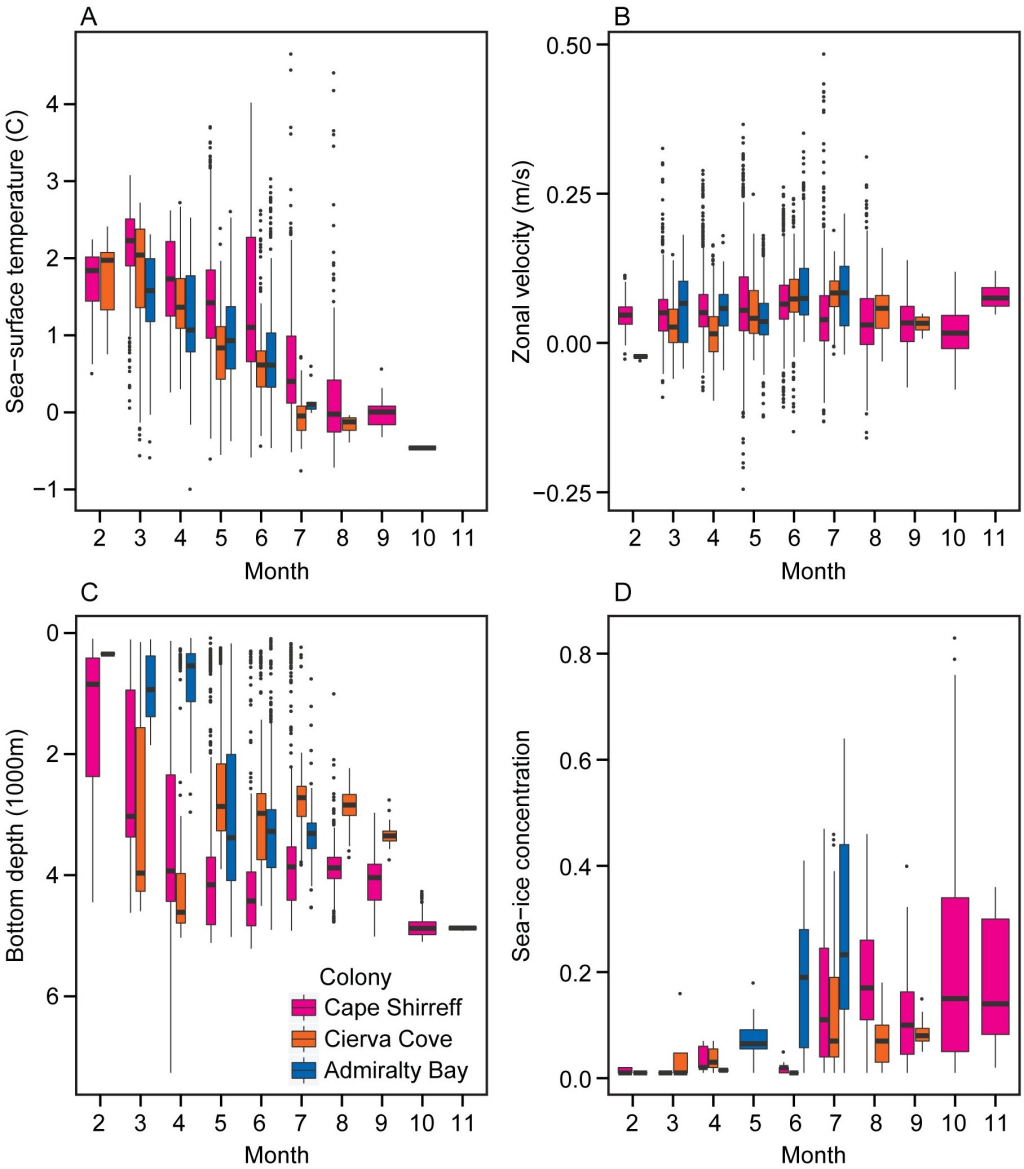
Boxplot of monthly distributions of environmental habitat covariates. Sea-surface temperatures (A), surface currents (B), bottom depths (C) and sea-ice concentrations (D) encountered within the habitat utilization distributions of chinstrap penguins originating from different colonies near the northern Antarctic Peninsula. Groupings are based on colony of origin.

Outbound migration during March through June occurred between the PF and the SACCF, with several individuals exhibiting the longest eastward or westward migrations tracking the SACCF (Fig 2). The monthly movements of ‘local’ birds are reproduced at higher resolution (Fig 3) to illustrate their off-shelf movement by May and occupancy of deep-water habitats primarily to the west and north of the South Shetland Islands through mid-winter. All movements from each colony were generally in slow, eastward flowing ice-free water with temperatures ≤2°C (Figs 2–4 and S3 Fig) irrespective of migration direction (S4 Fig). The environmental conditions encountered in the HUDs during the outbound migration for each colony differed over time (Fig 4 and S4 Fig). As winter progressed, birds from each colony occupied increasingly colder waters, but birds from Cape Shirreff occupied slightly warmer water than birds from the other colonies. Depths also decreased by month, but birds from Admiralty Bay remained over the shelf until through April. Thereafter, all birds were typically in deep ocean basins with median bottom depths exceeding 2500 m.

During mid-winter months (July through October) when sea ice extent was greatest, chinstrap penguins were distributed across a wide longitudinal range near the ice edge (Fig 2). As ice advanced, particularly between 120°W and 60°W, the birds abandoned areas near the SACCF and retreated north toward the PF. Mean monthly sea-ice concentrations in the HUDs were typically less than 40%, but denser sea ice did occur within the HUD (Fig 4). Residence time in ice-covered areas, however, was minor and represented less than 1% of the duration of the tracks based on the along-track matching of raw location estimates with daily SIC data. Such excursions into the marginal ice zone were characterized by daily SICs averaging 37.2 ± 0.04%, consistent with the monthly averages assessed for the HUDs. All birds continued to use pelagic areas south of the PF that were characterized by slow, eastward flowing water over deep ocean basins during mid-winter. Sea-surface temperatures beyond July were generally the coldest encountered during the year (Fig 4).

During the winter, individuals occupied areas from roughly 170°W to 25°W, spanning a distance of roughly 8000 km at 60°S. For the western-most observation, the nearest point of land was Cape Adare on the northwest coast of the Ross Sea, roughly 1080 km to the southwest. The nearest known chinstrap breeding colonies, in the Balleny Islands, are similarly 1100 km to the southwest (66.89°S, 163.6°E). The nearest point of land to the eastern-most observation was roughly 215 km to the southwest at Zavadoski Island (56.22°S, 27.57°W), in the South Sandwich Islands, home to the world’s largest known chinstrap breeding colony [43]. Thus, chinstrap penguins from the northern Antarctic Peninsula integrate a vast oceanic region that spans all chinstrap breeding areas in the western hemisphere.

**Fig 5:**
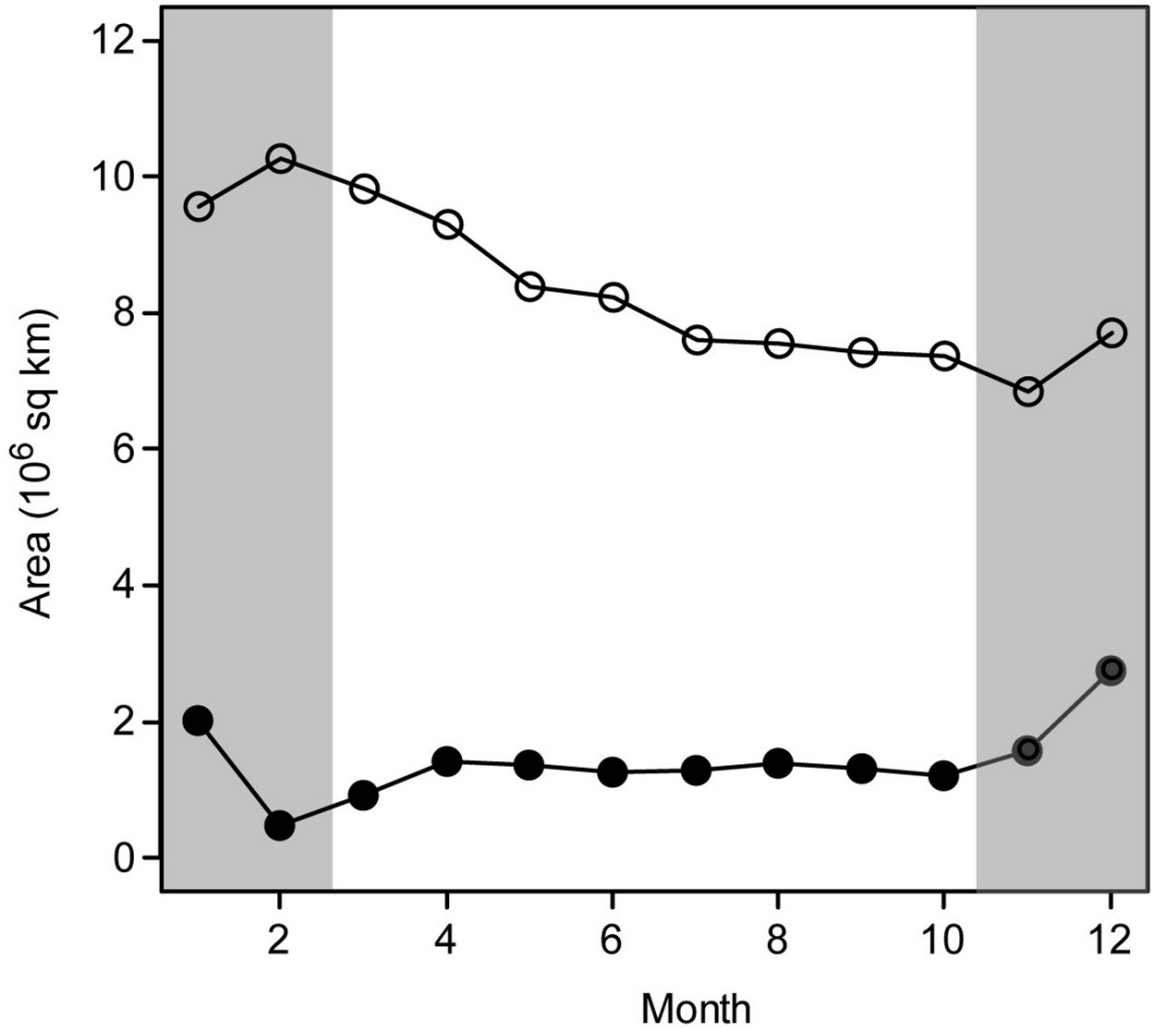
Area of open water and marginal ice zone habitats from 180° W to 0° W in 2017. Estimated areas of open-water habitat available (2°C isotherm to 5% sea-ice concentration; open circles) and marginal ice zone (MIZ; 5–50% sea ice concentration; closed circles) habitat and to chinstrap penguins during 2017. The breeding season is marked in gray.

Across this wide expanse of Southern Ocean, the birds generally remained in cold (≤2°C) waters south of the PF, even as sea ice advanced north (Fig 2). The open-water habitat area available to chinstrap penguins decreased through the winter of 2017 (Fig 5), driven largely by the northern advance of sea ice. The narrow area of the MIZ that was infrequently accessed by chinstrap penguins, however, remained largely constant throughout the winter, showing the greatest change in total area between December and February (Fig 5), when adult chinstrap penguins would typically be in coastal foraging areas near their breeding colonies.

**Fig 6:**
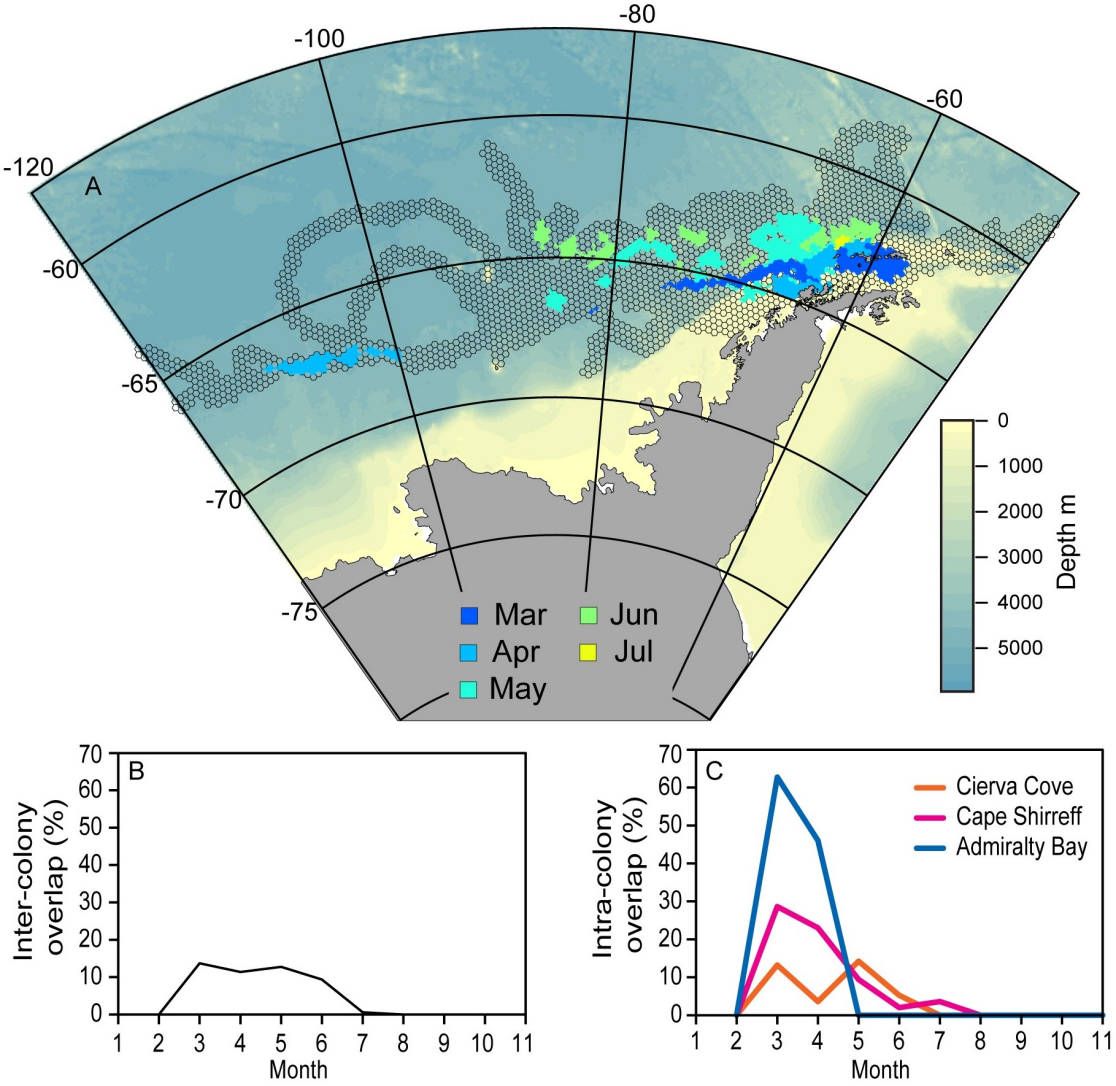
Map and indices of overlap. A) Areas of monthly inter-colony overlap from the three tagging locations. For reference, the background HUD (gray) is combined across all months and tagging locations. B) Monthly inter-colony overlap as a percentage of the total area occupied each month by birds from all colonies. C) Monthly intra-colony overlap as a percentage of the total area occupied each month by birds from a given colony.

**Fig 7:**
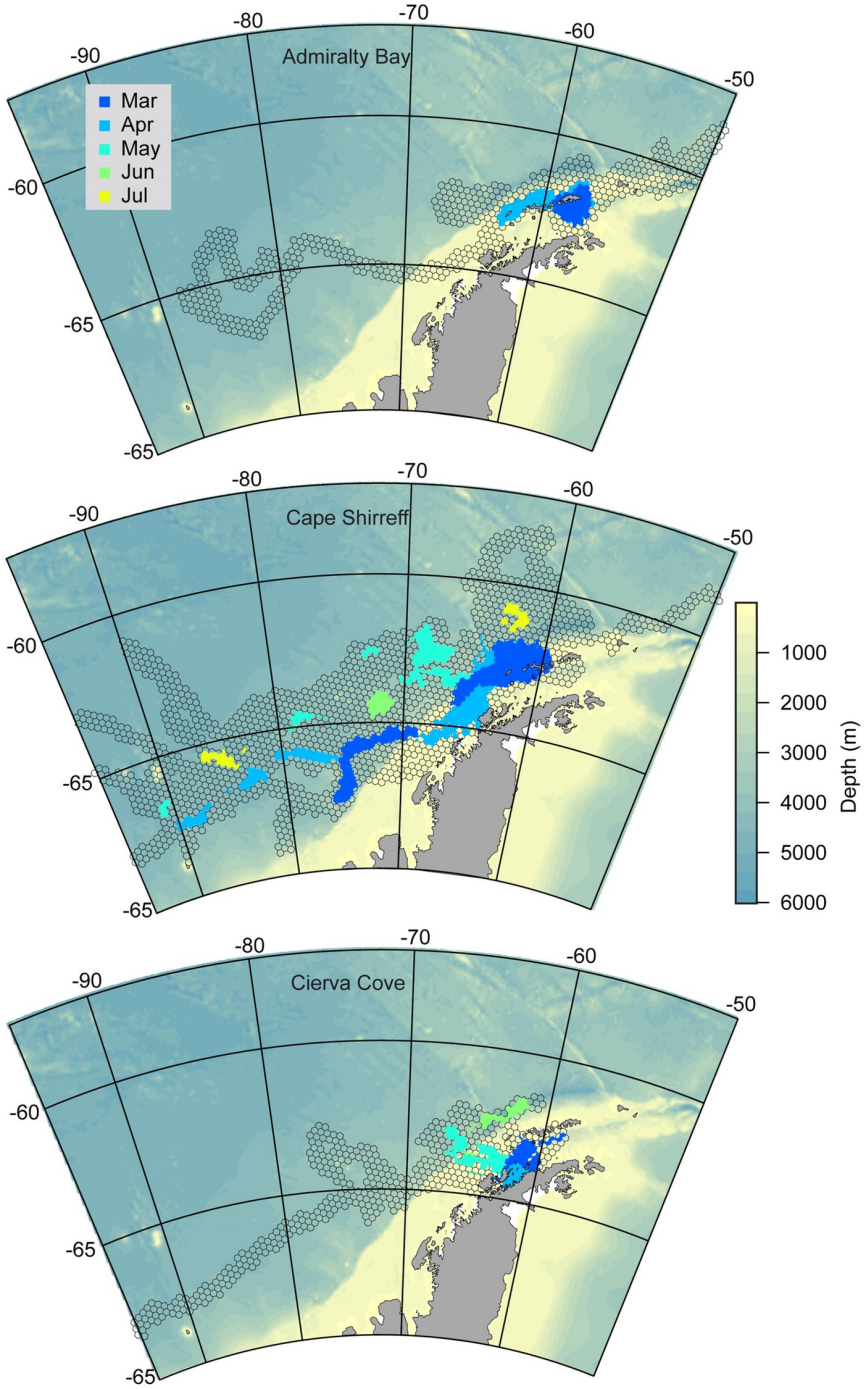
Intra-colony overlap maps. Monthly intra-colony overlap maps for individuals from Admiralty Bay, Cape Shirreff, and Cierva Cove. For reference, the background HUD (gray polygons) is the colony-specific total HUD for all months combined.

Despite the decrease in general habitat availability across the broad longitudinal range of areas that chinstrap penguins occupied, overlap of the monthly HUDs was low. Inter-colony overlap by birds from the three colonies was highest from March through June, covering roughly 10–13% of the total monthly HUD area each month (Fig 6). During this time, inter-colony overlap was present primarily in waters west of Antarctic Peninsula region and in a narrow movement corridor west of 100°W (Fig 6). Similarly, intra-colony overlap was low relative to the total monthly area occupied by birds from each tagging location, respectively (Figs 6c and 7). Birds from Cape Shirreff and Admiralty Bay exhibited higher overlap in March and April, but overlap declined as individuals continued their westbound or eastbound migrations to occupy more-distant habitats. For birds originating at Cape Shirreff, overlap occurred in remote areas along southern margins of the total HUD, indicating shared movement corridors for some individuals (Fig 7).

## Discussion

Tracking the winter migrations of adult and juvenile chinstrap penguins from multiple breeding locations within the northern Antarctic Peninsula region revealed shared east-west migration corridors along the SACCF but relatively little overlap of wintering areas within or across colonies. Rather, chinstrap penguins distributed themselves broadly between the sea ice edge and the PF, occupying progressively generally colder (<2° C), ice-free, and deeper physical habitats that spanned the western hemisphere of the Southern Ocean. Across this broad area, inter-colony and intra-colony overlap was generally low except in the months immediately following tagging over shelf areas in the vicinity of the study colonies. We interpret the patterns of overlap and wide distribution of birds across the Southern Ocean to suggest that the availability of suitable physical habitat for chinstrap penguins is widespread throughout the winter.

At a colony level, the tracking data support expectations that colony location influenced the general direction of travel during outbound migration [16]. For example, no animals were observed moving east from the Cierva Cove colony, while 20% of birds from Admiralty Bay moved east. Adherence to such colony-specific movement tendencies suggests that wintering areas may also differ by colony. The available data support this assertion on the basin-level scale, despite limited levels of intra-colony overlap of individuals. On the large scales of the local, eastward, and westward migration classifications, the proportions of penguins that remained local during winter varied by colony. Moreover, birds from the increasing population at Cierva Cove exhibited the highest proportion of local migrants, while birds from the sharply declining population at Cape Shirreff exhibited the lowest proportion of local migrants. This positive correlation raises a testable hypothesis that chinstrap penguin colonies in the Antarctic Peninsula region with increasing populations exhibit more local migration strategies. Winter conditions near the Antarctic Peninsula region, which are increasingly ice-free [44], yet characterized by relatively high krill densities [45], may benefit the penguins, and hence their colonies, that choose to remain local relative to birds that exhibit longer-distance movements. For example, the population size and breeding range of the non-migratory gentoo penguins (*P*. *papua*) are increasing in the Antarctic Peninsula region in contrast to their migratory congeners, the chinstrap and Adélie (*P*. *adeliae*) penguins [8]. Alternatively, we note that birds assigned to the local category exhibited the shortest average deployments (Table 2). The cause of such short deployments may arise from tag failure, tag loss, or animal death, which the available data cannot differentiate. The former two causes are not incompatible with the hypothesized benefit of local migration strategies, but early death in the local habitat would be contradictory. Irrespective of cause, an early loss of telemetry data within the local bin would inflate the proportion of birds assigned as local migrants. Correction of such bias may weaken the observed correlations with population trends and invalidate the hypothesized benefit of a local migration strategy. Clearly, further tracking studies will be necessary to resolve these uncertainties. Data from the Mapping Application for Penguin Populations and Projected Dynamics [46] suggest opportunity for such further study, as multiple chinstrap colonies with contrasting population trajectories exist throughout the Peninsula region (S5 Fig).

Nonetheless, the limited degree of spatial and temporal inter-colony and intra-colony overlap suggests that individual variations in movement are the primary factor driving the ultimate spatial patterns of habitat occupancy across the Southern Ocean during winter. From each colony, some adults and juveniles remained relatively local, while others rapidly moved thousands of kilometers along relatively narrow corridors, with individuals choosing unique stopover locations along the way. Such high levels of individual variability in movement patterns can make unambiguous identification of colony-specific wintering areas problematic [47]. Multi-year tracking data of chinstrap penguins from Cape Shirreff and Admiralty Bay [12,15] also exhibit winter movement patterns characterized by high levels of individual and inter-annual variation (S6 Fig). Moreover, the tracking data from 2017 fall within the scope of movements previously established by prior studies, particularly the winter tracking efforts from 2010 and 2011 [15]. Similar levels of variability in movement patterns across years occurred despite substantial variability in large-scale environmental indices that affect Southern Ocean processes such as the El Niño Southern Oscillation and sea-ice extent [48]. As with the tracking data, the broad-scale environmental indices of 2017 were intermediate to those from years with historical tracking data (S7 Fig). We interpret this high degree of variation in movement against the backdrop of environmental variability to suggest that chinstrap penguins are opportunistic during winter without clear preference for shared hotspots or unique colony-specific locations.

Despite the similarity of general physical habitats used by chinstrap penguins during winter, smaller-scale processes undoubtedly inform individual-level selection of foraging areas. For example, local hydrographic variation and frontal zones influence the distribution of foraging areas by individual penguins during the breeding season [24,49,50], and it is likely that winter foraging habitats are identified based on similar cues. For example, the prominent role of the SACCF in guiding outbound migration demonstrates the importance of large-scale frontal features for chinstrap migration generally, while inter-annual differences in foraging areas of chinstrap penguins during winter have been linked to spatiotemporal variability in local frontal features [12].

We assume that fine-scale habitat use during winter is also driven by heterogeneity in local prey distributions. However, such distributions are virtually unknown across the extent of areas occupied. A prior study of the stable isotopic niches occupied by chinstrap penguins during winter revealed elevated ^15^N signatures in tail feather tissues of chinstraps that migrated west [14] highlighting that prey availability varied longitudinally and included higher trophic-level prey items for migrants that move west into the Pacific sector of the Southern Ocean. Indeed, there is evidence for increased krill density around the Antarctic Peninsula during winter relative to summer months [45], suggesting the krill may be a relatively more-important resource for local migrants than for long-distance migrants. However, there remains no confirmation of diet composition from birds that reside in different winter habitats to resolve how prey distributions might influence such broad predator distributions or whether spatial differences in diet are related to divergent population trends across colonies.

Multi-colony studies of some wide-ranging seabird species have highlighted convergence of individuals from multiple colonies on relatively small-scale, specific locations [5,51]. Such convergence to foraging hotspots during winter provides an opportunity for conservation and spatial planning, for example to inform management of mineral resource extraction or fishing in the hotspot. Here, we find little evidence of such convergence to hotspots. Rather, post-molt adult and fledgling movements indicate occupancy of broad swaths of available habitat, including shelf areas. The shelf areas, particularly in the Bransfield Strait and north of the South Shetland Islands, generally held the areas of highest overlap. It is noteworthy that such overlap was observed from March into May (Fig 7) for adults and juveniles, noting that most juvenile tracks ceased shortly after deployment. The early loss of juvenile tracking data leaves an important gap in our understanding of how this life stage uses coastal areas and whether such habitats affect their survival and recruitment. We suggest that continued research to better map juvenile penguin movements to evaluate habitat effects on overwinter survival and recruitment [10] is a key need for current management efforts in the region.

Conservation and management efforts for chinstrap penguins may therefore be prioritized by focusing on these coastal foraging habitats over the shelf of the Antarctic Peninsula region. There, spatial and functional overlap with the Antarctic krill fishery occurs [15], and colony-specific exposure to gradients in climate warming and localized fishing effort may be critical factors in shaping population responses [19]. Furthermore, foraging effort in penguins generally tends to be highest around the molt period and before the breeding season [52,53]. These critical periods occur when chinstrap penguins occupy coastal foraging areas. Data on foraging movements over the shelf have the potential to inform fisheries management efforts [15,54], identify marine Important Bird and Biodiversity Areas [55], and inform marine spatial management efforts [56,57]. Indeed, tracking data were used to help develop agreed priority areas for conservation in the development of a proposed marine protected area in the western Antarctic Peninsula region [58].

The long-term decline in abundance of chinstrap penguins is the predominant trend observed at multiple locations in the Antarctic Peninsula and Scotia Sea region [8], though examples of colonies with increasing trends exist (S6 Fig) [8,21,59,60]. The negative regional trend throughout much of the chinstrap range, however, is of concern [19] given the pace and magnitude of climate change [59,61] and the recent developments of the fishery for Antarctic krill [62]. During winter, a mixing of individuals across the Southern Ocean from different breeding colonies in the Peninsula region would expose all colonies to a variable, yet shared environmental experience. Such shared environmental experience helps align the regional responses of chinstrap penguins around the Antarctic Peninsula.

## Supporting information

S1 Fig Distances attained from tagging locations during deployment for adult and juvenile chinstrap penguins tagged near the northern Antarctic Peninsula region. (PDF)

S2 Fig Raw tracks and duration of deployment for each track from each colony. (PDF)

S3 Fig Monthly maps of penguin positions, sea-surface current, and sea-ice concentrations. (PDF)

S4 Fig Boxplot of monthly distributions of environmental habitat covariates. (PDF)

S5 Fig Chinstrap breeding sites with increasing or decreasing populations. (PDF)

S6 Fig Multi-year tracking data of chinstrap penguins during winter from Admiralty Bay and Cape Shirreff. (PDF)

S7 Fig Environmental indices for years with chinstrap winter tracking data. (PDF)

S1 Data Zipped file with satellite telemetry data and historical nest census data. (ZIP)
