# Supplementary material for: Individual variation in migratory movements of chinstrap penguins leads to widespread occupancy of ice-free winter habitats over the continental shelf and deep ocean basins of the Southern Ocean
Source: PLoS One. 2019 Dec 10;14(12):e0226207. doi: 10.1371/journal.pone.0226207 (PMC6903731; doi:10.1371/journal.pone.0226207)

S1 Fig. Distances attained from tagging locations during deployment for adult and juvenile chinstrap penguins tagged near the northern Antarctic Peninsula region. Panel A is referenced to colony of origin. Panel B is referenced to directional movement bins (west, local, or east). Panel C demonstrates, for each tagging individual, the distance attained from its tagging location over time. Note the lack of directional movement tendencies in the 'local' category across the first 15 weeks of deployment when the 500km limit is used to define directional categories.

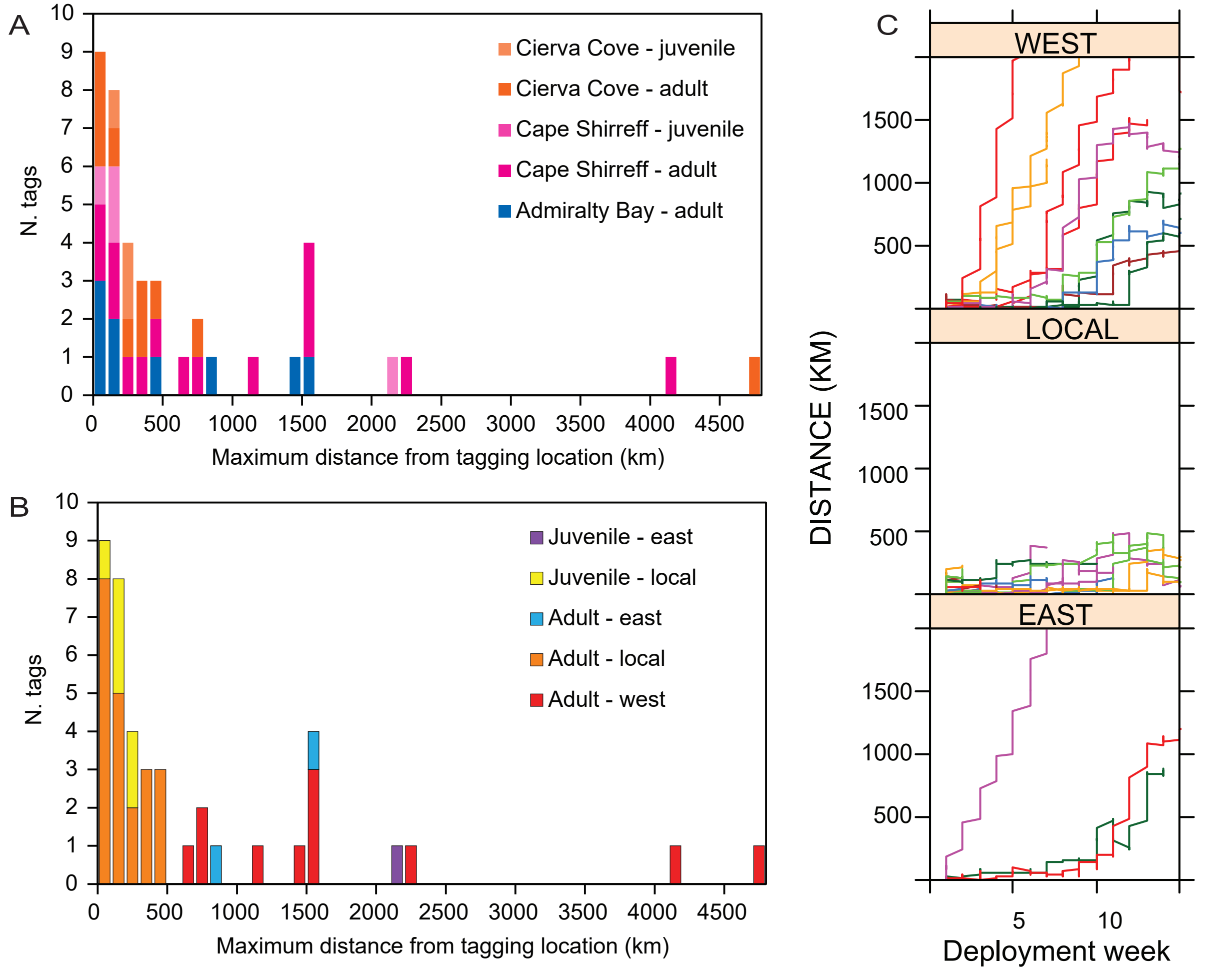

Supplement: S1 Fig — (PDF) [file pone.0226207.s001.pdf]
