# Supplementary material for: Individual variation in migratory movements of chinstrap penguins leads to widespread occupancy of ice-free winter habitats over the continental shelf and deep ocean basins of the Southern Ocean
Source: PLoS One. 2019 Dec 10;14(12):e0226207. doi: 10.1371/journal.pone.0226207 (PMC6903731; doi:10.1371/journal.pone.0226207)

S2 Fig. **Raw tracks and duration of deployment for each track from each colony.** Raw tracking data and a histogram of track duration from each colony indicating west-bound, local, and east-bound tracks of adult and juvenile chinstrap penguin tagged near the northern Antarctic Peninsula.

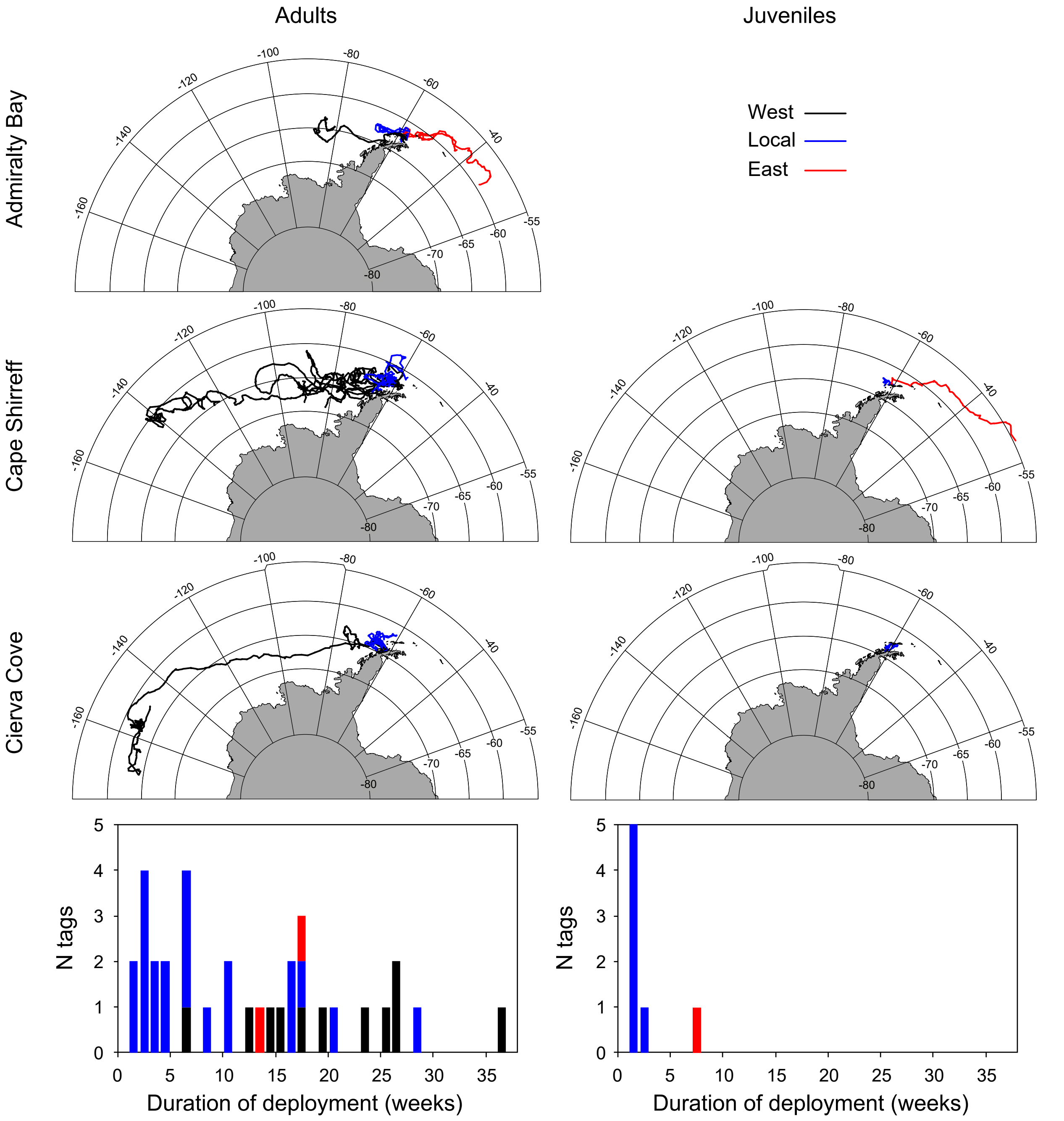

Supplement: S2 Fig — (PDF) [file pone.0226207.s002.pdf]
