# Supplementary material for: Individual variation in migratory movements of chinstrap penguins leads to widespread occupancy of ice-free winter habitats over the continental shelf and deep ocean basins of the Southern Ocean
Source: PLoS One. 2019 Dec 10;14(12):e0226207. doi: 10.1371/journal.pone.0226207 (PMC6903731; doi:10.1371/journal.pone.0226207)

S3 Fig. Monthly maps of penguin positions, sea-surface current, and sea-ice concentrations. Monthly at-sea locations of chinstrap penguins originating from Admiralty Bay (blue), Cape Shirreff (magenta), and Cierva Cove (orange) overlaid on mean monthly sea-surface currents and mean monthly sea-ice concentrations, March – October, 2017. The Polar Front (thick solid line), southern Antarctic circumpolar current front (thin solid line) and southern boundary of the Antarctic circumpolar current (thin dashed line) are plotted for reference.

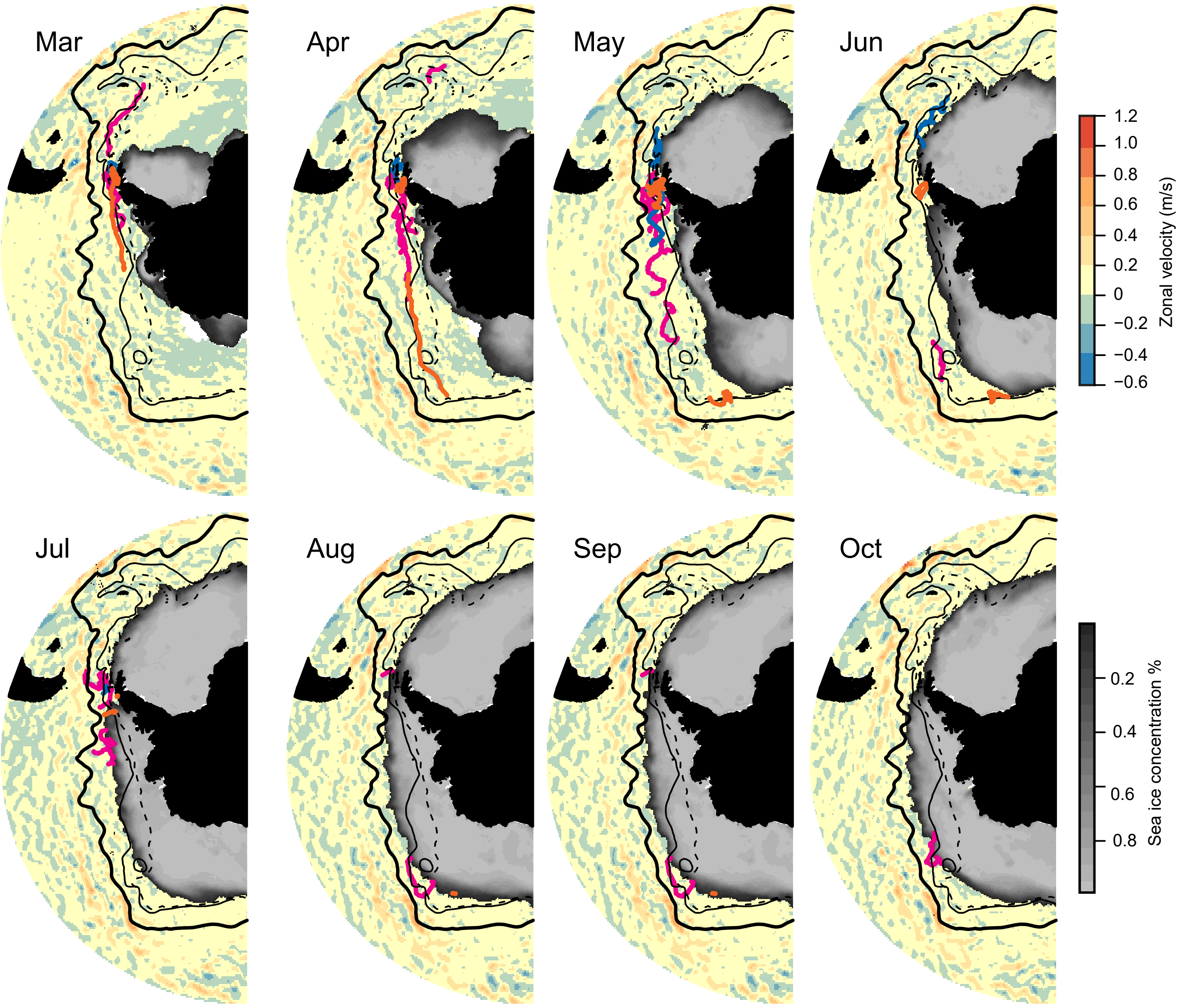

Supplement: S3 Fig — (PDF) [file pone.0226207.s003.pdf]
