# Supplementary material for: Individual variation in migratory movements of chinstrap penguins leads to widespread occupancy of ice-free winter habitats over the continental shelf and deep ocean basins of the Southern Ocean
Source: PLoS One. 2019 Dec 10;14(12):e0226207. doi: 10.1371/journal.pone.0226207 (PMC6903731; doi:10.1371/journal.pone.0226207)

S4 Figure. Boxplot of monthly distributions of environmental habitat covariates. Sea-surface temperature (A), surface currents (B), bottom depths (C) and sea-ice concentrations (D) encountered within habitat utilization distributions of chinstrap penguins originating from different colonies near the northern Antarctic Peninsula. Groupings based on direction of travel.

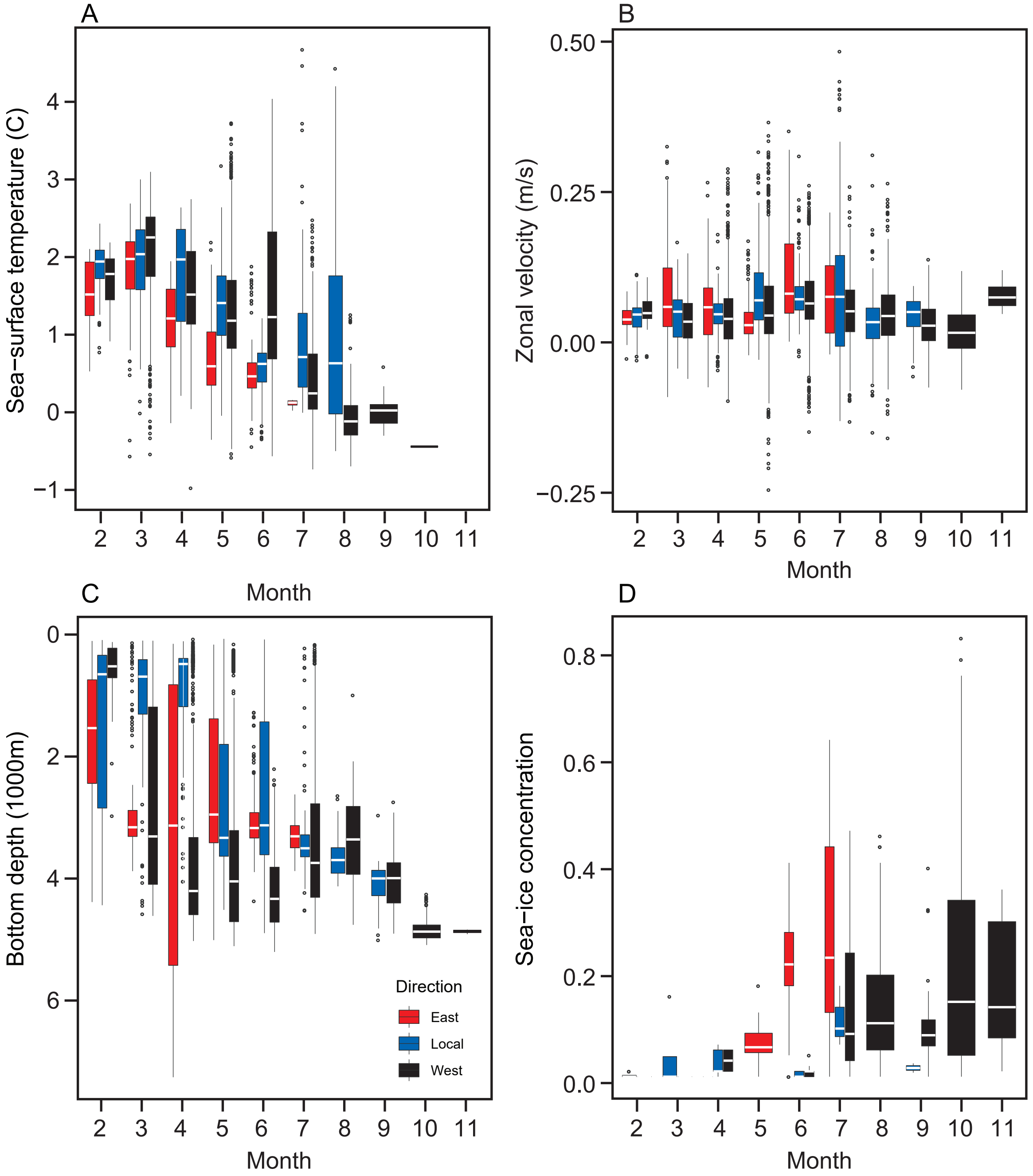

Supplement: S4 Fig — (PDF) [file pone.0226207.s004.pdf]
