# Supplementary material for: Individual variation in migratory movements of chinstrap penguins leads to widespread occupancy of ice-free winter habitats over the continental shelf and deep ocean basins of the Southern Ocean
Source: PLoS One. 2019 Dec 10;14(12):e0226207. doi: 10.1371/journal.pone.0226207 (PMC6903731; doi:10.1371/journal.pone.0226207)
Supplement: S5 Fig — (PDF) [file pone.0226207.s005.pdf]

S5 Fig. Chinstrap breeding sites with increasing or decreasing populations. Locations of chinstrap penguin breeding sites in the Antarctic Peninsula region with either increasing (green triangles) or decreasing (red triangles) populations based on linear trends calculated for sites with ≥4 years of count data. Symbol size for the triangles is scaled to the magnitude of the trend. Sites with less <4 years of count data were excluded. The locations of the main study colonies are highlighted for reference. All count data acquired from the Mapping Application for Penguin Populations and Projected Dynamics [1] at http://www.penguinmap.com/ [accessed 7 October 2019].

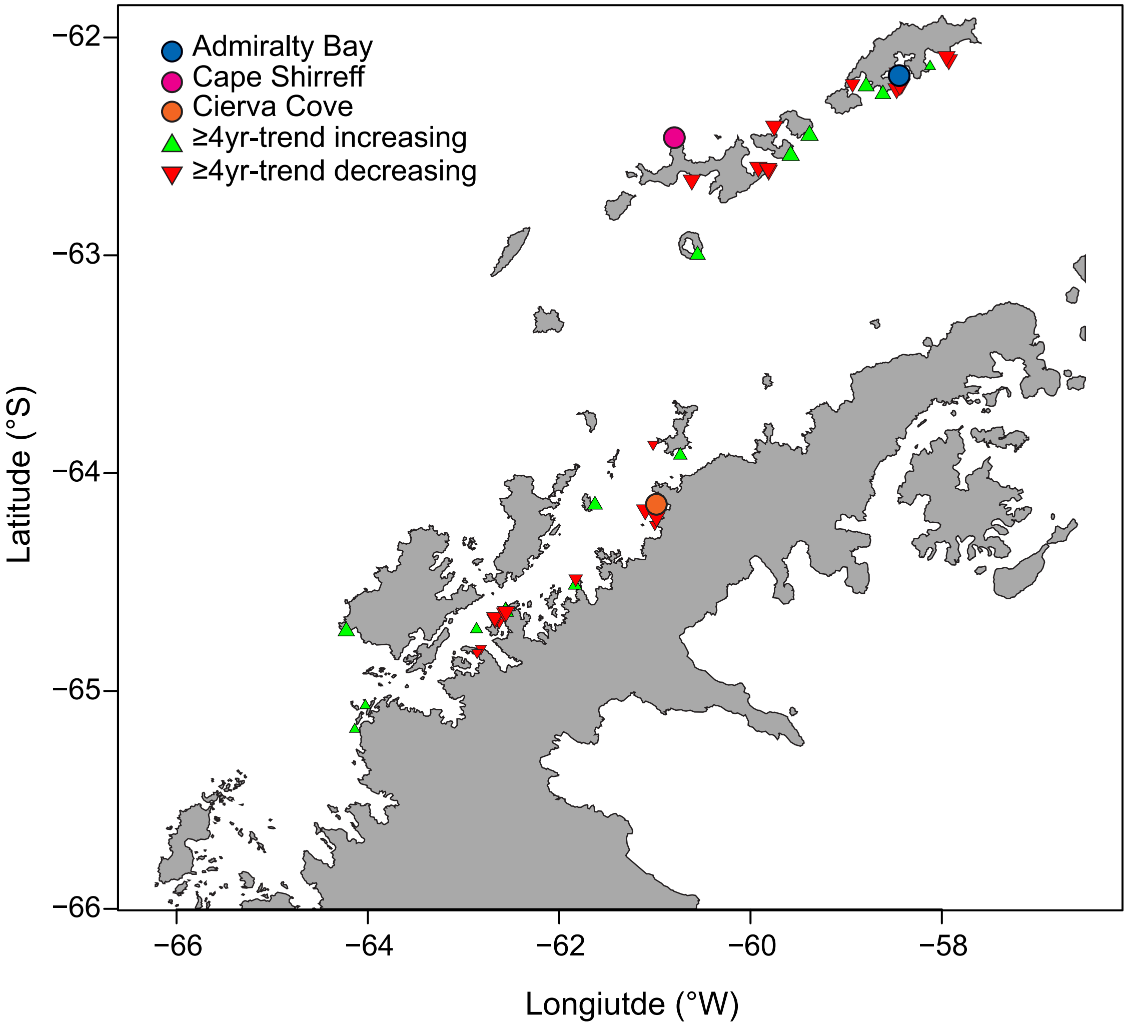

References

1. Humphries G, Naveen R, Schwaller M, Che-Castaldo C, McDowell P, Schrimpf M, et al. Mapping Application for Penguin Populations and Projected Dynamics (MAPPPD): data and tools for dynamic management and decision support. Polar Rec. 2017; 53:60-166. doi:10.1017/S0032247417000055.
