# Supplementary material for: Individual variation in migratory movements of chinstrap penguins leads to widespread occupancy of ice-free winter habitats over the continental shelf and deep ocean basins of the Southern Ocean
Source: PLoS One. 2019 Dec 10;14(12):e0226207. doi: 10.1371/journal.pone.0226207 (PMC6903731; doi:10.1371/journal.pone.0226207)
Supplement: S6 Fig — (PDF) [file pone.0226207.s006.pdf]

S6 Fig. Multi-year tracking data of chinstrap penguins during winter from Admiralty Bay and Cape Shirreff. Winter movements of chinstrap penguins have been tracked multiple times at Cape Shirreff and Admiralty Bay and have been reported previously [1,2], except for seven tracks from 2006 that are provided here.

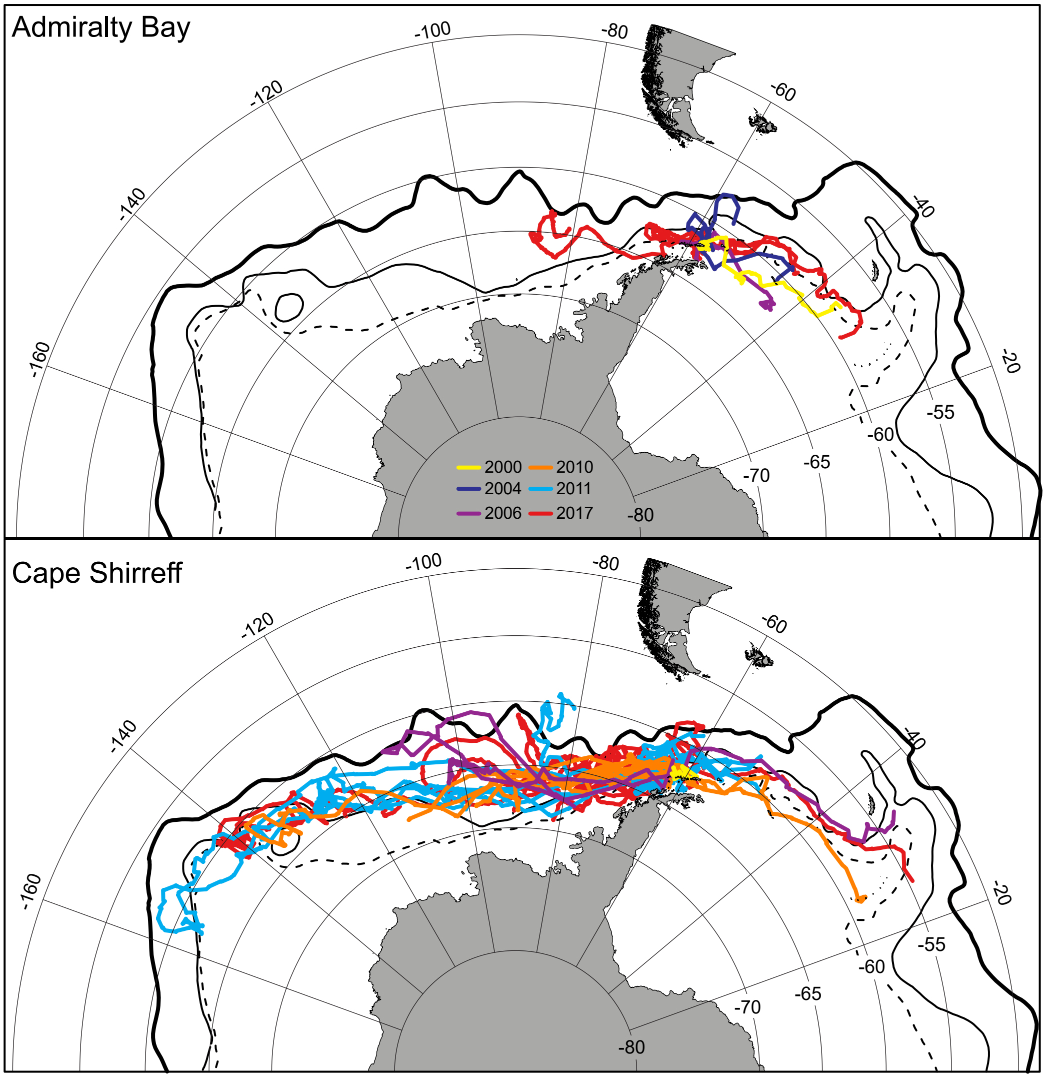

References

1. Trivelpiece WZ, Buckelew S, Reiss CS, Trivelpiece SG. The overwinter distribution of chinstrap penguins from two breeding sites in the South Shetland Islands of Antarctica. Polar Biol. 2007; 30:1231-1237.

2. Hinke JT, Cossio AM, Goebel ME, Reiss CS, Trivelpiece WZ, Watters GM. Identifying risk: concurrent overlap of the Antarctic krill fishery with krill-dependent predators in the Scotia Sea. PLoS ONE. 2017; 12(1): e0170132. doi: 10.1371/journal.pone.0170132
