# Supplementary material for: Individual variation in migratory movements of chinstrap penguins leads to widespread occupancy of ice-free winter habitats over the continental shelf and deep ocean basins of the Southern Ocean
Source: PLoS One. 2019 Dec 10;14(12):e0226207. doi: 10.1371/journal.pone.0226207 (PMC6903731; doi:10.1371/journal.pone.0226207)
Supplement: S7 Fig — (PDF) [file pone.0226207.s007.pdf]

S7 Fig. Environmental indices for years with chinstrap winter tracking data. Plots of the Oceanic Nino Index [1] and sea-ice extent [2] for selected months (March, June, September) of 2000, 2004, 2006, 2010, 2011, and 2017.

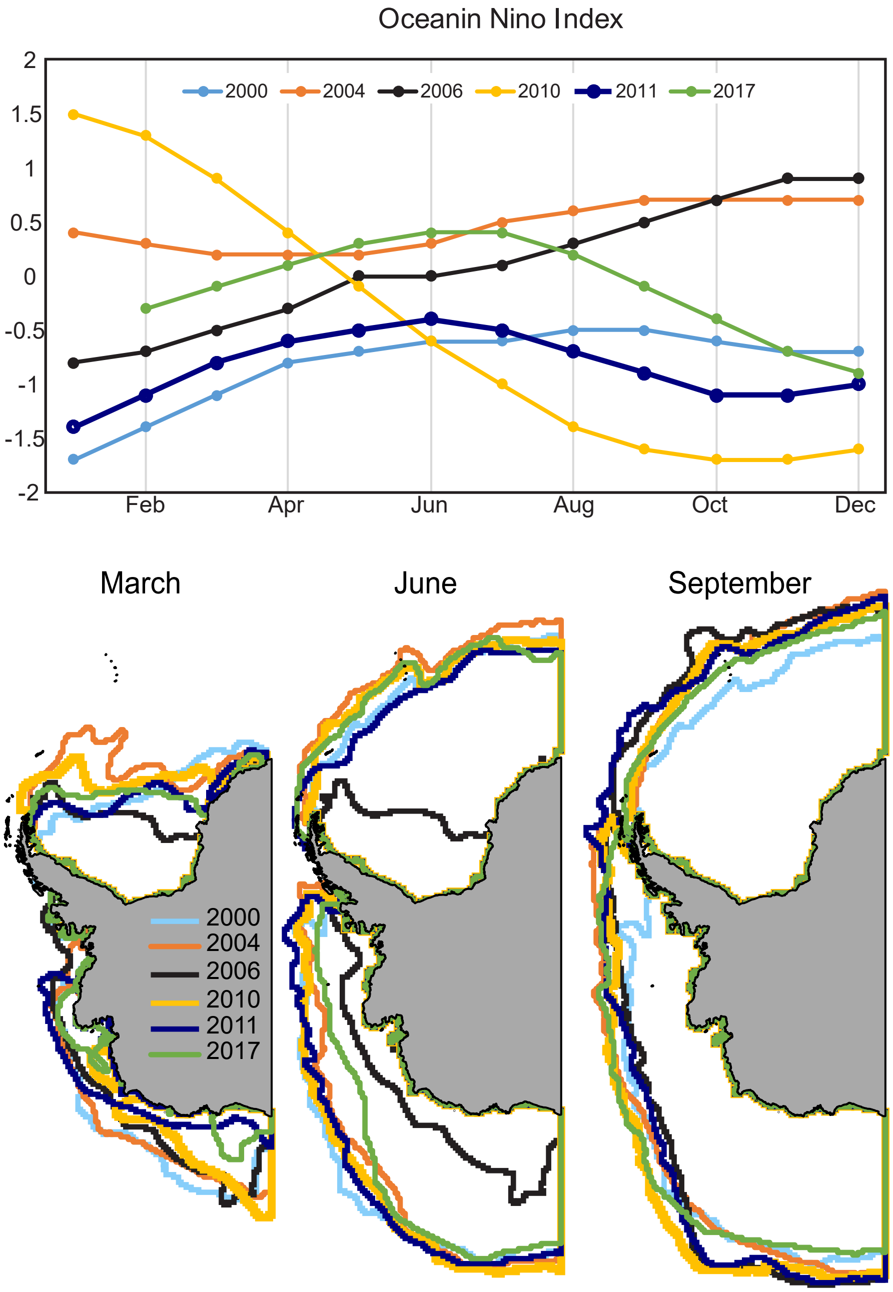

References

1. NOAA/National Weather Service; 2019 [cited 2019 Oct 8] Oceanic Niño Index [Internet]. Available from: https://origin.cpc.ncep.noaa.gov/products/analysis_monitoring/ensostuff/ONI_v5.php

2. National Snow and Ice Data Center; 2019 [cited 2019 Oct 8]. Data and Image Archive [Internet]. Available from: https://nsidc.org/data/seaice_index/archives
